# HSF-YOLO: A Multi-Scale and Gradient-Aware Network for Small Object Detection in Remote Sensing Images

**DOI:** 10.3390/s25144369

**Published:** 2025-07-12

**Authors:** Fujun Wang, Xing Wang

**Affiliations:** 1 School of Geomatics, Liaoning Technical University, Fuxin 123000, China; 2 Key Laboratory of Preparation and Application of Environmental Friendly Materials, Chinese Ministry of Education, Jilin Normal University, Changchun 130103, China; 3 School of Information Science and Engineering, Linyi University, Linyi 276000, China; wangxing@lyu.edu.cn

**Keywords:** remote sensing images, small object detection, multi-scale feature representation, attention mechanism

## Abstract

Small object detection (SOD) in remote sensing images (RSIs) is a challenging task due to scale variation, severe occlusion, and complex backgrounds, often leading to high miss and false detection rates. To address these issues, this paper proposes a novel detection framework named HSF-YOLO, which is designed to jointly enhance feature encoding, attention interaction, and localization precision within the YOLOv8 backbone. Specifically, we introduce three tailored modules: Hybrid Atrous Enhanced Convolution (HAEC), a Spatial–Interactive–Shuffle attention module (C2f_SIS), and a Focal Gradient Refinement Loss (FGR-Loss). The HAEC module captures multi-scale semantic and fine-grained local information through parallel atrous and standard convolutions, thereby enhancing small object representation across scales. The C2f_SIS module fuses spatial and improved channel attention with a channel shuffle strategy to enhance feature interaction and suppress background noise. The FGR-Loss incorporates gradient-aware localization, focal weighting, and separation-aware constraints to improve regression accuracy and training robustness. Extensive experiments were conducted on three public remote sensing datasets. Compared with the baseline YOLOv8, HSF-YOLO improved mAP@0.5 and mAP@0.5:0.95 by 5.7% and 4.0% on the VisDrone2019 dataset, by 2.3% and 2.5% on the DIOR dataset, and by 2.3% and 2.1% on the NWPU VHR-10 dataset, respectively. These results confirm that HSF-YOLO is a unified and effective solution for small object detection in complex RSI scenarios, offering a good balance between accuracy and efficiency.

## 1. Introduction

Remote sensing, powered by the proliferation of high-resolution satellite and aerial imagery, has become indispensable in different applications, including environmental monitoring, precision agriculture, disaster assessment, and urban planning [1,2]. Within this context, small object detection in remote sensing images plays a critical role in identifying fine-grained targets such as ships, vehicles, aircraft, and man-made facilities [3,4]. According to the commonly used Common Objects in Context (COCO) definition, small objects are defined as those with pixel sizes smaller than 32 × 32 [5]. These targets in RSIs are often extremely challenging to detect because of their small size, varying scales, arbitrary orientations, severe occlusions, and complex background interference [6,7]. Deep learning (DL), especially convolutional neural networks (CNNs), has become the mainstream approach for improving SOD performance in such complex scenarios, owing to their strong feature representation and generalization capabilities [8,9].

With the rapid advancement of DL, detection frameworks based on CNNs have achieved substantial success in SOD tasks within RSIs. Generally, these methods can be classified into two main types: two-stage and one-stage detectors [10]. Two-stage detectors, such as NACAD [11], CRPN-SFNet [12] and Gaussian-based R-CNN [13], operate by first generating region proposals, and then followed by object classification and bounding box regression. These methods, often adapted from Faster R-CNN [14,15] and enhanced with specialized modules, are known for their high detection accuracy, especially in complex scenes. However, two-stage methods often suffer from high computational overhead and slow inference speed. To overcome these limitations, one-stage detection methods have gained traction due to their real-time processing advantages. These methods regress object classes and bounding box locations directly from the input image, thereby eliminating the region proposal stage and reducing inference latency [16,17]. Typically, the YOLO (You Only Look Once) series has shown remarkable promise for SOD in RSIs due to its excellent balance between speed and accuracy [18,19]. Moreover, the YOLO series, such as YOLOv3 [20], YOLOv5 [21], YOLOX [22], YOLOv8 [23], and YOLOv10, has evolved through continuous architectural innovations to adapt to SOD in remote sensing imagery. These include improvements in backbone design, feature fusion modules, attention mechanisms, and so on [24,25,26,27,28]. These advances demonstrate the flexibility of the YOLO framework in adapting to the challenges of detecting small, occluded, and densely distributed targets in RSIs. More recently, YOLOv8 has emerged as a new benchmark within the YOLO series, incorporating the C2f module, decoupled detection heads, and an anchor-free assignment strategy. It has also been widely adopted in remote sensing SOD tasks, where it demonstrates competitive performance across various complex scenarios including occlusion, dense distribution, and scale variation [29,30,31].

Building upon these architectural advancements, a growing number of studies have proposed improved YOLOv8-based frameworks tailored for remote sensing SOD. These efforts aim to address the core issues of weak small object representation, dense spatial distributions, object occlusion, background clutter, and significant scale variation. The improvements span several key aspects, including backbone and neck structure redesign, multiscale feature enhancement, attention-guided fusion, and loss function optimization, among others [32,33,34,35]. The method in [2] improves YOLOv8 by adding a shallow feature layer and an upsampling branch to retain small object details. A 3D convolution-based SSFF module further enhances multiscale feature alignment, reducing missed detections from scale variation and dense layouts. SMA-YOLO [36] improves YOLOv8 by adding a small-object-specific detection layer and a CEF module with CDW-EMA attention to improve multiscale perception and background suppression. It also replaces the detection head with a dynamic head to reduce missed detections under occlusion and dense object distributions. RNAF-YOLO [37] enhances YOLOv8 by introducing RepFocalNet to strengthen multiscale feature extraction and ASCPA attention to highlight small and occluded targets. It also proposes FIS loss to focus training on hard samples, reducing missed detections in dense and cluttered scenes. Despite these advances, most existing YOLOv8-based models optimize isolated components such as shallow layers or attention blocks, lacking a unified strategy across the entire detection pipeline. Feature loss, semantic misalignment, and limited gradient feedback for low-Intersection over Union (low-IoU) samples still lead to missed and false detections, especially under dense, occluded, and multi-scale conditions.

To address the above-mentioned issues, a comprehensive detector HSF-YOLO, jointly enhancing feature extraction, attention integration, and regression precision for robust SOD in RSIs, is proposed in this paper. HSF-YOLO is built upon the YOLOv8n architecture and integrates specialized modules designed to handle weak semantics, dense distribution, and background interference associated with small objects in complex scenes. The main contributions of this paper are summarized as follows:

- We propose a Hybrid Atrous Enhanced Convolution (HAEC) module that captures both global semantic and fine-grained features via parallel atrous and standard convolutions, improving multi-scale feature extraction for small object detection;
- We design a Spatial–Interactive–Shuffle attention (C2f_SIS) module that integrates spatial and enhanced channel attention with shuffle operations to enhance discriminative representation and suppress background interference;
- We introduce a Focal Gradient Refinement Loss (FGR_Loss) that incorporates gradient-aware weighting, separation constraints and focal scaling to boost localization precision, especially under occlusion and object drift;
- Extensive experiments conducted on three public RSI datasets demonstrate the effectiveness and generalizability of the proposed HSF_YOLO, achieving consistent improvements over YOLOv8 and other state-of-the-art detectors.

The remainder of this paper is organized as follows. Recent advances in SOD for RSIs is reviewed in Section 2, with a focus on YOLO-based detectors. The overall architecture of the proposed HSF-YOLO is described in Section 3, including the design of the HAEC module, the C2f_SIS attention mechanism, and the FGR-Loss function. Section 4 presents the experimental details and provides a comprehensive performance comparison and ablation analysis to demonstrate the effectiveness of each module. Finally, conclusions and future directions are presented in Section 5.

## 2. Related Work

With the increasing demand for high-precision remote sensing applications, SOD in RSIs has emerged as a critical yet challenging task. Kinds of factors such as low resolution, scale variation, dense object distribution, and complex background interference significantly hinder accurate detection. Although two-stage detectors have been adapted for SOD in RSIs and achieved promising results, they typically incur substantial computational overhead and suffer from limited inference speed. In contrast, one-stage detectors provide a more favorable trade-off between accuracy and efficiency. Their end-to-end architecture and real-time performance make them particularly suitable for remote sensing scenarios. Accordingly, this section focuses on reviewing recent advances in one-stage detection approaches, with emphasis on improvements in network architecture, attention mechanisms, multi-scale feature fusion, and loss function design.

FPSOD [17] addresses SOD in RSIs based on a soft-thresholding filtering FPN (STF-FPN) with an attention-based soft-thresholding filter module (ASTFM) inserted between upsampling layers to suppress redundant features and enhance semantic salience. Its detection head comprises classification, localization, and centerness branches, optimized with focal, PR-GIoU, and centerness losses, achieving robust detection of small, dense targets in cluttered scenes. EFR-ACENet [38] focuses SOD in complex backgrounds and weak semantic features through explicit feature reconstruction (EFRM) and adaptive context enhancement (ACEM), combined with a composite loss including classification, confidence, and SIoU-based localization guides training to recover details, improve discrimination, and refine bounding box accuracy. DConvTrans-LGA [16] introduces a hybrid CNN-Transformer framework that combines dynamic convolution for spatial detail extraction and local self-attention to preserve fine-grained features. In addition, a residual pyramid network enhances multiscale fusion, while a combined classification and localization loss improves small object detection in occluded, low-confidence and densely distributed scenarios. SFSANet [39] focuses on scale and semantic consistency by introducing a Semantic Fusion (SF) module, a Spatial Location Attention (SLA) module for suppressing background noise, and a Scale Adaptability (SA) module leveraging dilated convolutions to capture multi-scale contextual details. A composite loss integrating classification, confidence, and SIoU localization terms further improves convergence and box accuracy for small, dense objects in complex scenes. CoF-Net [40] uses a progressive coarse-to-fine framework comprising a feature adaptation branch (CoF-FA) that refines features using spectral embedding and nonlocal modulation and a sample assignment branch (CoF-SA) that progressively selects training samples with geometric and semantic constraints, which collectively improve detection robustness for small, densely arranged objects in complex scenes. Together, these works illustrate an evolution from spatial detail enhancement to context-aware feature fusion and progressive optimization, reflecting a holistic effort to address the problems of SOD in complex remote sensing environments.

Due to their end-to-end architecture, real-time performance, and balance between accuracy and efficiency, YOLO-based methods have become increasingly popular in SOD tasks in complex remote sensing scenes. FFCA-YOLO [28] builds upon YOLOv5 by introducing three lightweight modules to improve SOD performance in occlusion and low-light conditions. The Feature Enhancement Module (FEM) is used for local contextual enrichment and discriminative enhancement of small objects against the background; the multiscale Feature Fusion Module (FFM) is applied to strengthen semantic consistency, and the Spatial Context Aware Module (SCAM) is presented to achieve global contextual interactions across channels and space. SCM-YOLO [25] also extends YOLOv5 by addressing spatial detail loss and insufficient global perception. It integrates the SPID module to enhance spatially localized information during downsampling, the CBCC module for adaptive block- and channel-level reweighting to improve multiscale feature fusion, and the MAGI module for suppressing background interference and enhancing object-background separability through local–global attention and residual connections. In [41], a YOLOv7-based remote sensing SOD method enhances the backbone with a stride-free convolution module to reduce spatial information loss, integrating a convolutional triplet attention (CTAM) and a contextual transformer (CoT) modules for local and global context refinement to effectively distinguish small objects in complex backgrounds, along with the MPDIoU loss to improve convergence speed and localization precision. MTGS-YOLO [42] further extends YOLOv7 by incorporating an M-Transformer to capture local and global context and reduce small object information loss, a GELAN module for lightweight multiscale feature extraction, and a SCAM module for spatial-channel attention refinement, addressing background clutter and small object occlusion. SOD-YOLOv10 [43] improves YOLOv10 by introducing the TransBone backbone for adaptive local–global information integration, AA-GFPN for aggregated attention-based multiscale feature interactions and background noise suppression, and the AFP-IoU loss function for faster convergence and improved localization accuracy of small and obscured targets, achieving robust and precise detection in complex scenes. These continuous improvements highlight the growing potential of YOLO-based methods, paving the way for further advancements with YOLOv8 as the latest and most promising extension [31,44].

YOLOv8 has rapidly become the mainstream architecture for SOD in RSIs, thanks to its advanced backbone design, robust feature representation, and highly flexible detection head. MFF-YOLOv8 [29] introduces a multi-scale feature fusion framework with an HFFP module using cross-scale connections and learnable weights to preserve fine-grained features, an RFS module for dynamic noise suppression and refinement, and a HEMA module for local–global context learning to reduce background interference. An Inner-WIoU loss enhances localization precision by combining distance-based penalties with scale-adaptive regression, addressing low-IoU issues for small targets. In [30], a lightweight SOD algorithm based on YOLOv8 is proposed to address feature loss and background clutter. It introduces a SOD layer for detail retention, an SSFF module for robust multi-scale fusion, and a novel HPANet that removes redundant layers while enhancing feature aggregation with Zoomcat-based operations, which collectively improve detection precision and speed for small objects in occluded and cluttered remote sensing environments. YOLO-GE [10] addresses small object feature loss and dense target detection with a G-HG block combining ghost convolution and CSP for enhanced multi-scale feature extraction, an improved E-SimAM attention mechanism for low-resolution feature learning, and a SOD head to detect dense small targets, which improve SOD accuracy and robustness in complex remote sensing scenarios with occlusion, low resolution and multi-scale. YOLO-SSP [33], based on YOLOv8, uses a lightweight SPD-Conv module to preserve fine-grained spatial information, adds a SOD layer for rich shallow feature extraction, and applies a PYSAM mechanism with hierarchical pooling and feature fusion to highlight critical spatial features and suppress irrelevant information. These combined improvements boost detection performance and robustness, especially for small and occluded objects. Finally, RNAF-YOLO [37] introduces a RepFocalNet module for deep multi-scale feature learning, an ASCPA module to strengthen small target feature representation and suppress background noise, and a Focal Inner SIoU loss function to dynamically adjust sample weights, focusing on hard-to-detect small targets and improving localization accuracy. However, existing YOLOv8-based methods still focus on optimizing isolated components and lack a unified strategy across the entire detection pipeline. As a result, issues like feature loss, semantic misalignment, and limited gradient feedback for low-IoU samples persist, particularly in dense, occluded, and multi-scale scenarios.

Recent DETR-based methods such as RT-DETR and DINO-DETR have demonstrated impressive performance in general object detection tasks due to their end-to-end design and powerful global modeling capabilities. EMSD-DETR [45] introduces a small object sensitive pyramid for cross-scale feature fusion that effectively fuses local spatial coordinate information with global contextual information to avoid missing details of different feature layers, and the Focaler-Shape-IoU to expedite convergence and enhance detection precision for difficult samples. DINO [46] improves over previous DETR-like models in performance and efficiency by using a contrastive way for denoising training, a mixed-query selection method for anchor initialization, and a look-forward-twice scheme for box prediction. However, these methods typically require high computational resources and long training times, making them less suitable for real-time or resource-constrained remote sensing applications. In contrast, YOLOv8n offers a better balance between speed, accuracy, and hardware efficiency, making it a strong baseline for small object detection in complex RSIs. Therefore, this work builds upon YOLOv8n and enhances its detection capability through three proposed modules.

However, most existing methods focus on optimizing individual modules, such as feature enhancement or attention guidance, yet lack a unified and systematic design framework. To address these issues, this paper proposes a holistic and collaboratively optimized detection framework, HSF-YOLO, which systematically enhances three key aspects: feature encoding, attention fusion, and regression precision. Building upon the YOLOv8n architecture, HSF-YOLO integrates three tailored modules: a Hybrid Atrous Enhanced Convolution module for global context capture and fine-grained detail preservation; a Spatial–Interactive–Shuffle attention module for improved spatial and channel attention fusion and noise suppression; and a Focal Gradient Refinement Loss for better convergence and precise localization of small, low-IoU targets. These integrated innovations enable HSF-YOLO to achieve excellent performance in detecting small objects under complex remote sensing scenarios.

## 3. Methodology

### 3.1. Overall Network Structure

**Figure 1:**
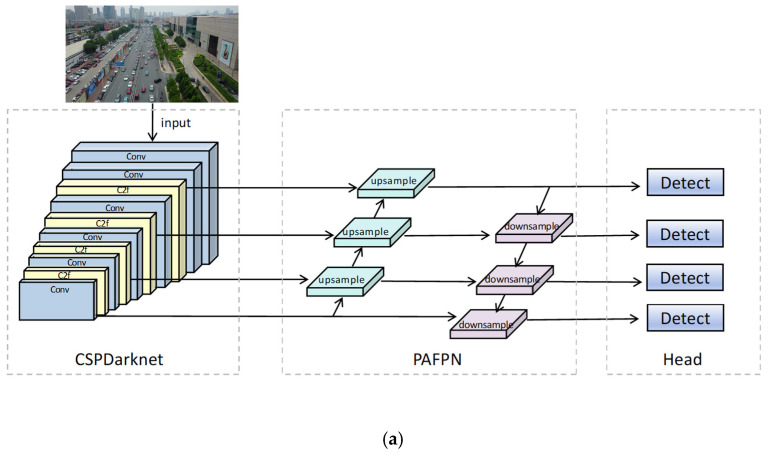

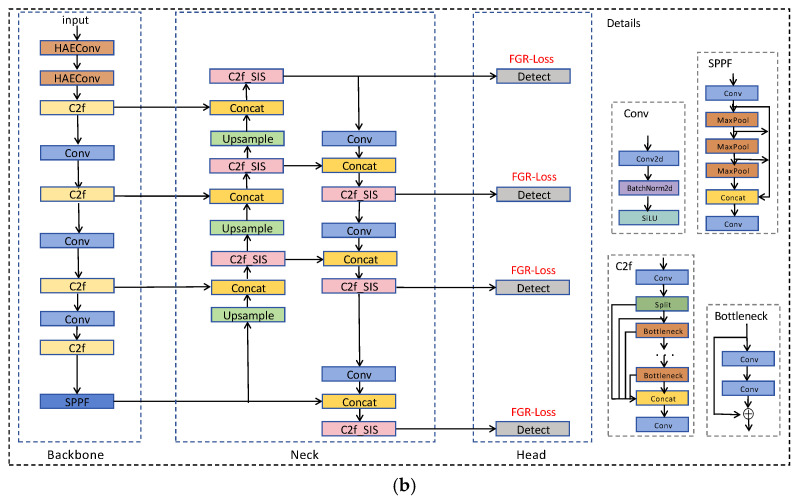
(**a**) Detection pipeline of YOLOv8; (**b**) The overall architecture of HSF-YOLO, including HAEC-enhanced backbone, C2f_SIS-augmented neck, gradient-aware FGR-Loss, and multi-head detection structure.

This paper proposes a novel detection framework named HSF-YOLO, built upon the YOLOv8 architecture, to effectively address the problems of SOD in RSIs, including large scale variation, dense object distribution, severe occlusion, and complex backgrounds. The overall architecture and core components of HSF-YOLO are illustrated in Figure 1. HSF-YOLO integrates three customized modules: HAEC, C2f_SIS, and FGR-Loss, each targeting a specific challenge commonly encountered in remote sensing-based SOD. The HAEC module, embedded in the backbone, combines standard and atrous convolutions in parallel to enrich multi-scale contextual information and preserve fine-grained local features. This enhancement significantly improves the representation of small objects, particularly those with scale variations or degraded edges. In the neck, the C2f_SIS module is employed to improve mid- and high-level feature interaction. It introduces efficient spatial and channel attention mechanisms, alongside channel shuffle operations, to refine semantic discrimination and suppress irrelevant background interference. This design enhances sensitivity to small and densely distributed objects in cluttered scenes. To further address the difficulty of detecting extremely small targets, an additional high-resolution detection layer is introduced. This layer operates on finer-scale feature maps to improve the localization and classification of small targets that are often overlooked by conventional detection heads. On the optimization side, we propose FGR-Loss, a novel regression loss function that replaces conventional IoU-based formulations. It enhances localization accuracy and training stability by combining gradient-aware learning, focal weighting for class imbalance, and a separation-aware penalty to mitigate prediction clustering in densely populated regions.

### 3.2. HAEC Module

**Figure 2:**
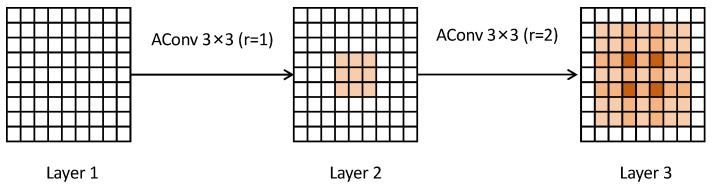
Schematic diagram of the cascade structure of multi-layer atrous convolutions. The first layer employs a 3 × 3 atrous convolution with a dilation rate r = 1, and the output feature map is followed by a second layer of 3 × 3 atrous convolution with r = 2. The orange regions represent the effective weights of the convolution kernels, and the white regions are the interpolated holes.

RSIs often contain densely distributed and variably scaled targets, coupled with complex background interference, which make it difficult for conventional convolutional backbones to extract sufficient semantic features, especially for small or occluded objects. Usually, atrous convolution is used to expand the receptive field without increasing the number of parameters (as shown in Figure 2). However, its use alone can introduce gridding artifacts and weaken the modeling of local textures, particularly in dense or occluded scenes. Thus, the HAEC module is designed to address this problem, which combines standard and dilated convolutions in a parallel structure to balance global context capture and local feature preservation.

**Figure 3:**
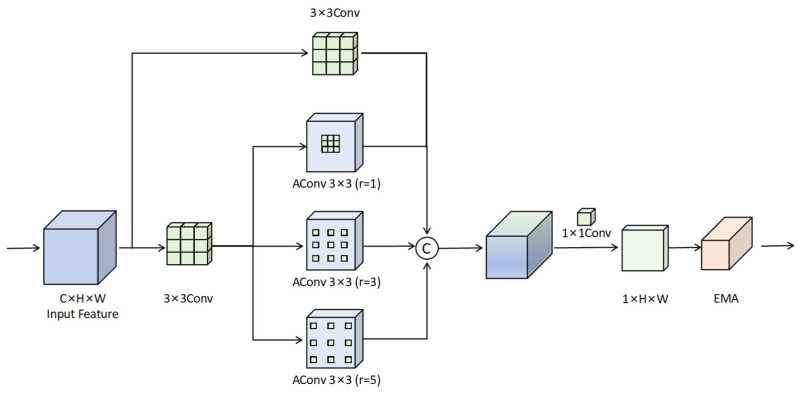
The detailed structure of HAEC, which combines multi-branch atrous convolutions and attention to enhance contextual information for small object detection.

As illustrated in Figure 3, given an input feature map $X\in R^{C\times H\times W}$, the main branch applies three parallel atrous convolutions with dilation rates r = {1,3,5}, formulated as:

(1) $$F_{dilated}=Concat(Conv_{3\times 3}^{d=1}(X),Conv_{3\times 3}^{d=3}(X),Conv_{3\times 3}^{d=5}(X))$$

where $Conv_{3\times 3}^{d=r}(\cdot )$ denotes a 3 × 3 convolution with dilation rate r. Meanwhile, an auxiliary branch with a standard convolution extracts local spatial details:

(2) $$F_{local}=Conv_{3\times 3}^{d=1}(X)$$

These two branches are then concatenated:

(3) $$F_{fused}=Concat(F_{dilated},F_{local})$$

Then, a 1D convolution $Conv_{1\times 1}$ is applied to reduce channel dimensionality and encourage inter-channel interaction:

(4) $$F_{main}=Conv_{1\times 1}(F_{fused})$$

**Figure 4:**
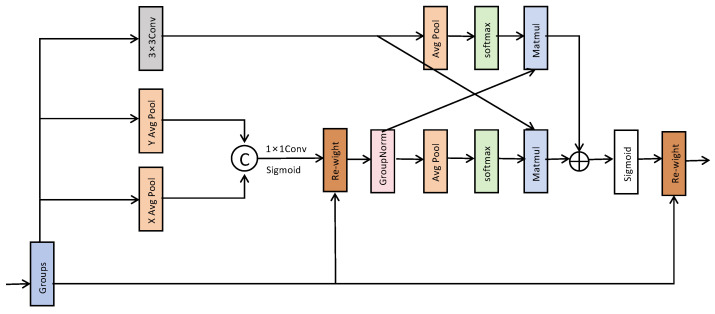
The detailed structure of the Efficient Multi-scale Attention (EMA) module.

Finally, the fused features are passed through the EMA module (as shown in Figure 4), which uses global pooling and channel reweighting to emphasize informative features:

(5) $$F_{EMA}=A_{EMA}(F_{main})$$

where $A_{EMA}(\cdot )$ denotes the attention weights computed by the EMA module. The HAEC module enhances the network’s ability to model both global semantics and fine spatial structures, significantly improving the robustness of detecting small and weakly represented targets.

### 3.3. C2f_SIS Module

**Figure 5:**
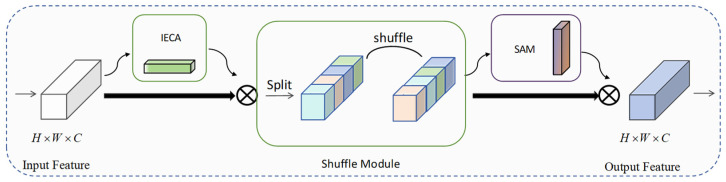
The architecture of the proposed C2f_SIS module, which integrates spatial attention, interactive channel attention, and channel shuffle operations to strengthen feature discrimination.

**Figure 6:**
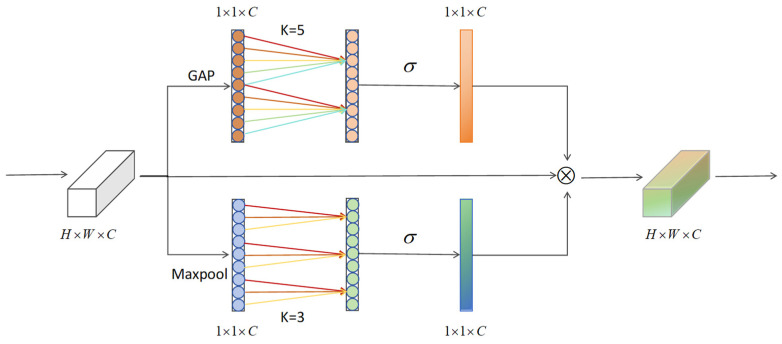
The IECA module structure for capturing refined channel dependencies.

**Figure 7:**
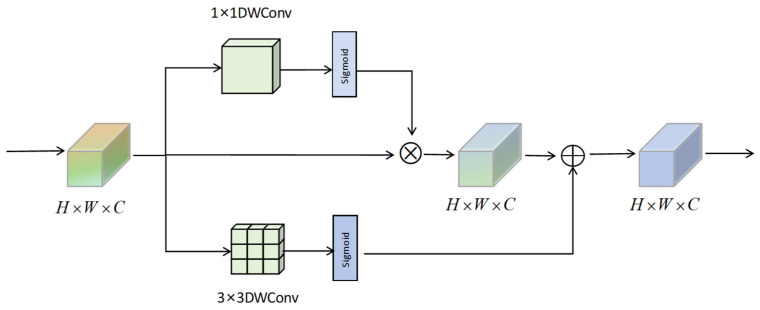
The structure of the SAMBlock for spatial attention enhancement.

To further improve the feature representation capability of YOLOv8 in SOD tasks for RSIs, a lightweight multi-dimensional attention module, Spatial–Interactive–Shuffle attention, is introduced in HSF-YOLO, as illustrated in Figure 5. This module aims to address multiple challenges faced in SOD in RSIs, including complex backgrounds, densely distributed targets, and significant intra-class variations. The C2f_SIS module integrates an improved efficient channel attention mechanism (IECA, as illustrated in Figure 6), a spatial attention sub-module (SAMBlock, as illustrated in Figure 7), and a channel shuffle mechanism, embedding them into the C2f structure of YOLOv8. While effectively improving the discriminative power of mid-to-high-level semantic features, this design maintains a low computational cost and demonstrates excellent accuracy and robustness, making it particularly suitable for SOD in real-world remote sensing scenarios.

The C2f_SIS module comprehensively enhances the feature perception of small objects in RSIs by collaboratively modeling in three dimensions: channel dimension, inter-channel group interaction, and spatial dimension. Specifically, the IECA module introduces a dual-branch structure that jointly extracts complementary channel information through global average pooling (GAP) and max pooling (GMP). This is followed by modeling with depthwise separable one-dimensional convolution, effectively capturing long-range dependencies between channels. Compared to traditional fully connected attention mechanisms, this approach maintains high efficiency while improving cross-scale channel selection capability. This module helps retain critical semantic details for SOD in RSIs, thereby enhancing HSF-YOLO’s sensitivity to small targets.

Given the input feature $X\in R^{C\times H\times W}$, the C2f_SIS module integrates three components. The module first aggregates channel-wise information using GAP and GMP:

(6) $$F_{avg}=GAP(X)$$

(7) $$F_{max}=GMP(X)$$

These pooled features are then processed by separate convolutional branches to capture different receptive field patterns. Subsequently, each branch undergoes a depthwise separable one-dimensional convolution.

(8) $$F_{avg\_conv}=DWConv_{1\times 1}(\sigma (Conv_{5\times 5}(F_{avg})))$$

(9) $$F_{\max\_conv}=DWConv_{1\times 1}(\sigma (Conv_{3\times 3}(F_{max})))$$

where σ(⋅) denotes a nonlinear activation function. Finally, the resulting attention weights are executed on the input feature map through element-wise multiplication:

(10) $$F_{IECA}=X⊗F_{avg\_conv}⊗F_{\max\_conv}$$

To further alleviate feature isolation caused by channel grouping, the C2f_SIS incorporates a channel shuffle mechanism after attention fusion. This strategy rearranges and redistributes channels to enable efficient cross-group information exchange without increasing computational overhead, enhancing feature diversity and cross-channel interaction. It facilitates multi-scale semantic information fusion and adapts well to common scale variations and dense target overlaps in RSIs.

To mitigate feature isolation caused by channel grouping, the reweighted features $F_{IECA}$ are divided into g groups and shuffled via a permutation operation S(⋅):

(11) $$F_{shuffle}=S(F_{IECA})$$

which facilitates efficient cross-group information exchange without additional computation. Finally, spatial attention weights are generated by combining two parallel branches: a lightweight 1 × 1 convolution followed by a 3 × 3 depthwise separable convolution:

(12) $$F=F_{shuffle}⊗\sigma (Conv_{1\times 1}(F_{shuffle}))⊕\sigma (Conv_{3\times 3}(F_{shuffle}))$$

In terms of spatial modeling, the SAMBlock module employs lightweight 1 × 1 convolution and Sigmoid activation to generate spatial attention maps, guiding the network to focus on discriminative spatial regions. Meanwhile, a parallel 3 × 3 depthwise separable convolution branch is introduced to strengthen local texture perception, making the attention map more sensitive to edge contours and other fine details. This spatial modeling mechanism effectively mitigates common issues in RSIs such as blurred boundaries, background interference, and illumination changes.

### 3.4. FGR-Loss

To enhance localization accuracy and improve training stability in SOD within complex remote sensing imagery, we propose a novel loss function termed Focal Gradient Refinement Loss. This composite loss addresses three core challenges that conventional object detection losses often overlook: vanishing gradients when the IoU is zero, foreground–background class imbalance, and prediction clustering in densely packed scenes. First, to resolve the gradient-vanishing problem frequently encountered in early-stage regression, FGR-Loss introduces a gradient-aware localization term that combines the Generalized Intersection over Union (GIoU) with Smooth L1 loss, formulated as:

(13) $$L_{loc}=\alpha \cdot (1-GIoU)+\beta \cdot SmoothL1(t_{pred},t_{gt})$$

where $t_{pred}$ and $t_{gt}$ denote the predicted and ground-truth bounding box parameters, and α and β are balancing weights. This design ensures that meaningful gradients are propagated even when predicted boxes do not initially overlap with ground truth, thereby improving convergence and localization precision. To mitigate the impact of class imbalance, particularly the dominance of easy negatives and background regions in remote sensing data, we incorporate a focal classification term which is defined as:

(14) $$L_{cls}=-\sum_{i}\alpha_{i}(1-p_{i}{)}^{\gamma }\log(p_{i})$$

where $p_{i}$ is the predicted class probability, $\alpha_{i}$ is a class-balancing factor, and γ is the focusing parameter that emphasizes learning from difficult examples such as small or partially occluded objects. Furthermore, to encourage better spatial distribution of predicted boxes in scenes with high object density, we introduce a separation-aware penalty term:

(15) $$L_{sep}=\sum_{\left(i,j\right)}\exp(-IoU(B_{i},B_{j}))\cdot \exp(-||c_{i}-c_{j}||)$$

where $B_{i}$ and $B_{j}$ are predicted bounding boxes and $c_{i}$, $c_{j}$ denote their respective center coordinates. This term penalizes excessive spatial overlap between predictions and encourages the network to produce well-separated detections. The final FGR-Loss is defined as a weighted combination of these components:

(16) $$L_{FGR}=\lambda_{1}L_{cls}+\lambda_{2}L_{loc}+\lambda_{3}L_{sep}$$

where $\lambda_{1}$, $\lambda_{2}$, and $\lambda_{3}$ are hyperparameters that control the contribution of each term. By jointly optimizing classification focus, regression stability, and prediction separability, the FGR-Loss function offers a practicable solution that significantly enhances the robustness and accuracy of SOD in remote sensing applications.

## 4. Experiments

### 4.1. Datasets

Three datasets are utilized in the experiments: the DIOR, the VisDrone2019 and the NWPU VHR-10 datasets.

The DIOR dataset, developed by Northwestern Polytechnical University, is a large-scale benchmark dataset specifically designed for object detection in RSI. It contains 23,463 images and 192,472 object instances. The dataset covers 20 diverse object categories, including airplanes, buildings, vehicles, and more. It is divided into three subsets in a 7:1:2 ratio, comprising 16,425 training images, 2346 validation images, and 4692 testing images. The resolution of each image is 800 × 800 pixels, and the spatial resolutions of images range from 0.5 m to 30 m. Compared with other datasets, the DIOR dataset offers advantages such as a large data scale, diverse object instance sizes, rich image variety, high inter-class similarity, and significant intra-class variation [10].

The VisDrone2019 dataset was collected by the AISKYEYE team from the Machine Learning and Data Mining Laboratory at Tianjin University, using drone-mounted cameras to capture images from an aerial perspective. It is a horizontal bounding box dataset designed for optical remote sensing object detection tasks. There are 288 video clips, 261,908 video frames and 10,209 static images in the dataset. It includes 12 different object categories, of which our experiments focus on 10 categories by excluding the “ignored regions” and “others” categories. Moreover, three subsets come from the dataset used for training, validation, and testing with a ratio of 7:1:2, containing 6471, 548, and 1610 images, respectively. Notably, the data was collected under various scenes, weather conditions, and lighting environments using multiple drone platforms, covering special cases such as scene visibility, object categories, and occlusion scenarios [10].

The NWPU VHR-10 dataset consists of high-resolution images selected from Google Earth and the Vaihingen databases. It contains ten object categories, with 650 images containing objects and 150 background images, totaling 800 images for spatial object detection. The dataset is divided into two subsets used for training and validation with a ratio of 8:2, including 520 and 130 images, respectively [39].

### 4.2. Implementation Details

The experiments are conducted on the DIOR, VisDrone2019, and NWPU VHR-10 datasets to verify the performance of HSF-YOLO. A unified data processing strategy is adopted to ensure fairness: the model optimizer is Stochastic Gradient Descent (SGD), whose initial learning rate is 0.01 and final learning rate is 0.001. The batch size is set to 8, and the entire training process involves 200 epochs. The rest of the configuration is left as the default configuration of the original YOLOv8 model.

The experimental platform is built upon the PyTorch deep learning framework, with hardware and software environments as follows: Windows 10 operating system, Intel(R) Core(TM) i9-10920X CPU @ 3.50 GHz, NVIDIA GeForce RTX 3090 24575MiB, Python version 3.10.14, PyTorch version 1.11.0, and CUDA version 11.3.1.

### 4.3. Evaluation Indicators

To evaluate the performance of HSF-YOLO, we use the following four indicators as evaluation criteria: precision (P), recall (R), mAP@0.5 and mAP@0.5:0.95. Respectively, mAP@0.5 and mAP@0.5:0.95 denote the average precision at the Intersection over Union thresholds of 0.5 and 0.5 to 0.95. In addition, FLOPs is a measure of computer hardware performance and algorithm complexity. The specific formula is as follows:

(17) $$P=\frac{TP}{TP+FP}$$

(18) $$R=\frac{TP}{TP+FN}$$

(19) $$AP=\int_{0}^{1}Pd(R)$$

(20) $$mAP=\frac{1}{N}\sum_{i=1}^{N}AP_{i}$$

where TP denotes the number of objects accurately detected, FP means the number of objects inaccurately identified, and FN represents the number of missed detection objects. AP is used to measure the performance of individual categories, while mAP measures the total performance across categories.

### 4.4. Experimental Results

To evaluate the overall effectiveness and generalization ability of HSF-YOLO, comparative experiments were conducted on three challenging public datasets: VisDrone2019, DIOR, and NWPU VHR-10. These datasets vary widely in terms of spatial resolution, object size distribution, background complexity, and annotation density. We compare HSF-YOLO with several classical and state-of-the-art detectors, including SSD, Faster R-CNN, YOLOv3/v5/v8, and recent advanced variants such as TA-YOLO, RNAF-YOLO, MFF-YOLOv8, and GLFE-YOLOX. The ison includes four commonly adopted performance indicators: precision (P), recall (R), mAP@0.5, and mAP@0.5:0.95. Results are shown in Table 1, Table 2 and Table 3.

**Figure 8:**
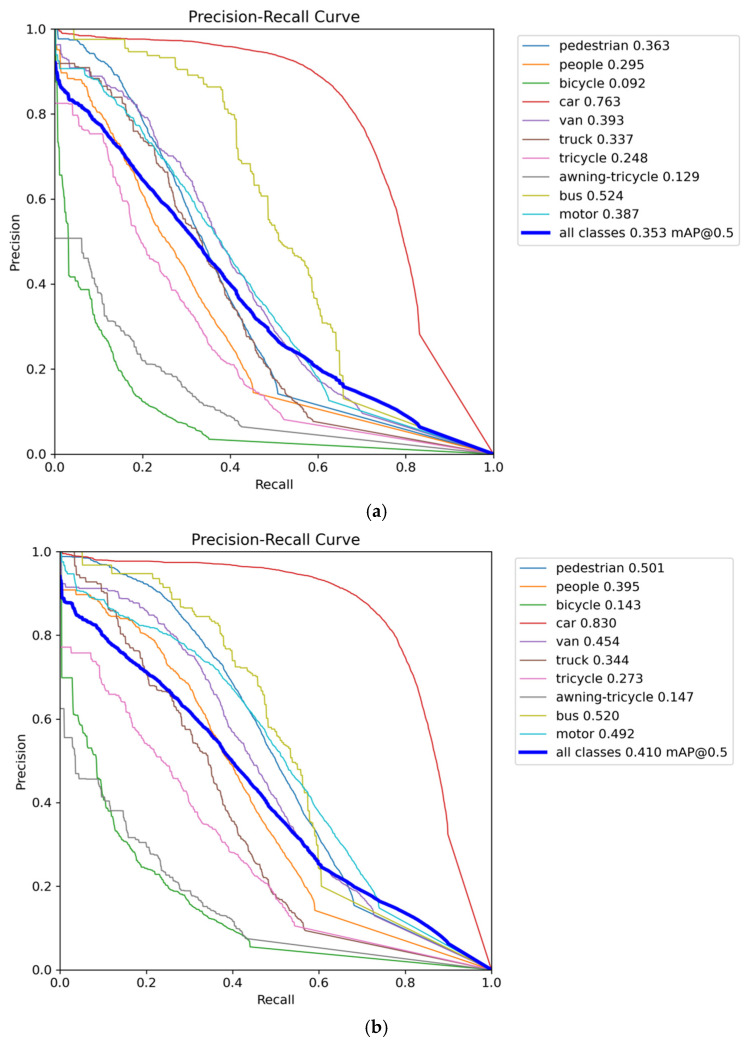
The precision–recall curves of different models on the VisDrone2019 dataset: (**a**) YOLOv8n, (**b**) HSF-YOLO.

**Figure 9:**
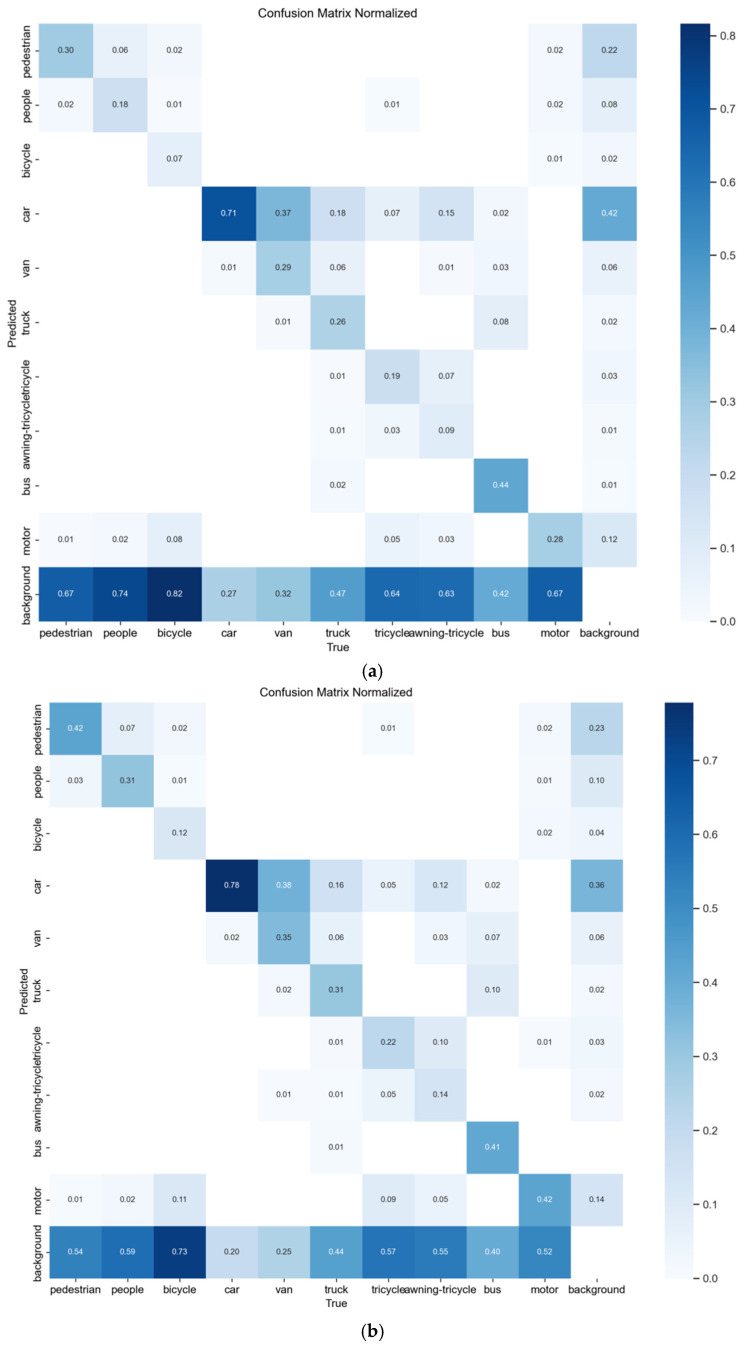
The confusion matrices of different models on the VisDrone2019 dataset: (**a**) YOLOv8n, (**b**) HSF-YOLO.

**Figure 10:**
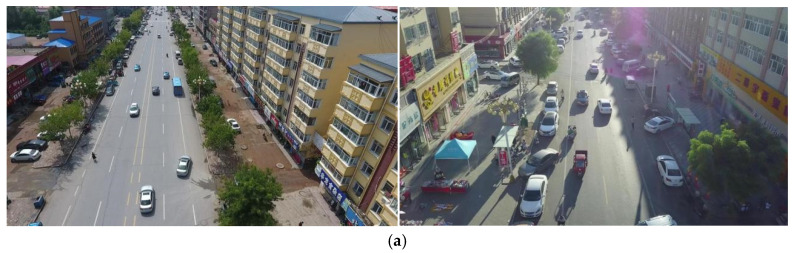

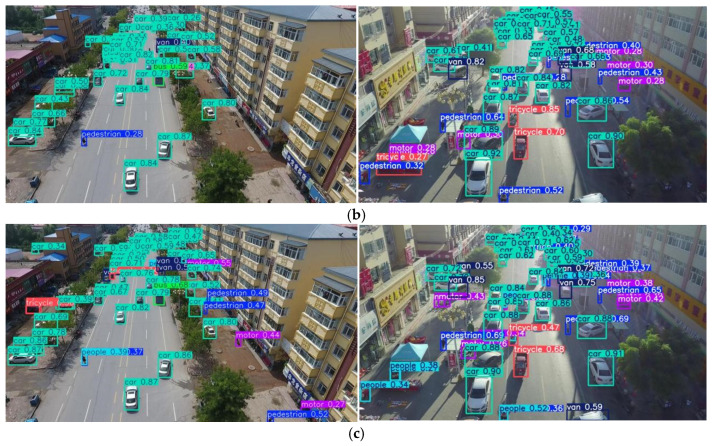
Visualization results of different models for the VisDrone2019 dataset: (**a**) original image, (**b**) YOLOv8n, (**c**) HSF-YOLO.

On the VisDrone2019 dataset, the proposed HSF-YOLO model achieves significant improvements across all metrics, reaching a precision of 51.6%, recall of 40.0%, mAP@0.5 of 41.0%, and mAP@0.5:0.95 of 24.6%. ed with the baseline YOLOv8n (46.1%, 35.1%, 35.3%, and 20.6%), HSF-YOLO achieves notable gains of 5.5%, 4.9%, 5.7%, and 4.0%, respectively, demonstrating improved feature representation and modeling capabilities for SOD and localization in RSIs. Table 1 shows the detailed performance ison between HSF-YOLO and other classical and state-of-the-art object detectors on the VisDrone2019 dataset. In ison with traditional detectors such as SSD and Faster R-CNN, which yield mAP@0.5:0.95 values of only 13.6% and 10.7%, respectively, it is evident that these early one-stage and two-stage detection methods encounter significant bottlenecks under remote sensing scenarios characterized by dense targets, large scale variations, and complex backgrounds. Furthermore, when compared to representative YOLO series models (YOLOv3, YOLOv5s, YOLOX, YOLOv8n), although these models achieve a certain balance between detection speed and accuracy, their improvements in mAP@0.5:0.95 remain limited in scenarios with densely distributed small objects and drastic scale changes, reflecting their inadequacy in handling complex remote sensing tasks. Moreover, HSF-YOLO maintains its performance advantage over several recently proposed advanced detectors, such as SCM-YOLO, MTGS-Yolo, YOLO-GE, FPSOD, TA-YOLO, RNAF-YOLO, and MFF-YOLOv8. Although these methods improve detection performance by incorporating attention mechanisms, multi-scale fusion structures, and novel regression losses, their mAP@0.5:0.95 values still fall short of the 24.6% achieved by HSF-YOLO. In particular, compared with the best-performing model TA-YOLO, HSF-YOLO improves by 0.6% in mAP@0.5:0.95 while also maintaining superior precision and recall. In addition, two recent DETR-based small object detection models, DFS-DETR and EMSD-DETR, which are built upon RT-DETR, achieve mAP@0.5:0.95 values of 23.1% and 23.0%, respectively. Despite leveraging the strengths of Transformer architectures, their performance remains inferior to that of HSF-YOLO. This demonstrates a more optimal balance between detection accuracy and object coverage, further validating the effectiveness of the proposed multi-module collaborative optimization strategy in SOD under complex remote sensing conditions. The comparative precision–recall curves and confusion matrices of different models on the VisDrone2019 dataset are depicted in Figure 8 and Figure 9, respectively. The visual detection results on this dataset are illustrated in Figure 10.

As shown in Figure 9, compared with the baseline model, the proposed model exhibits generally higher values along the diagonal of the confusion matrix, indicating a significant improvement in detection accuracy, particularly for vehicle- and pedestrian-related categories such as car, people, and motor. Meanwhile, the off-diagonal values have decreased overall, reflecting enhanced capability in distinguishing between similar categories, delineating object boundaries, and suppressing background interference.

Nevertheless, the off-diagonal values remain relatively high for certain categories, indicating that class confusion still exists. In the VisDrone dataset, this issue is particularly evident among semantically similar categories such as awning-tricycle, tricycle, van, as well as pedestrian and people. These targets often appear similar in size, shape, and color from an aerial perspective, making them more prone to confusion—especially in complex scenes with dense object distribution and severe occlusion. In addition, the dataset contains a large number of low-resolution small objects, which makes it difficult for the model to learn sufficiently discriminative features, ultimately leading to misclassification. This further highlights the challenges of fine-grained small object detection in real-world remote sensing imagery.

Figure 10 also presents typical cases of false positives and missed detections. For example, in Figure 10b,c, pedestrian targets are simultaneously labeled as both “person” and “pedestrian”, and confusion also arises between vehicles and trucks. These detection errors are primarily caused by a combination of factors. First, the dataset contains many semantically similar categories (such as car and van, pedestrian and people), which easily lead to class confusion. Second, due to dense object distribution and severe occlusion, the model struggles to accurately distinguish object boundaries, resulting in duplicate detections or missed detections. Additionally, distant or extremely small targets lack sufficient distinguishable features, making them difficult for the model to detect effectively. Shadows, billboards, and water reflections in complex backgrounds can also be mistakenly identified as real targets. Finally, inconsistencies or omissions in some sample annotations further impair the model’s learning process. These issues are especially prominent in remote sensing images, where multi-scale variation, low resolution, and complex backgrounds significantly limit detection accuracy and model robustness.

**Figure 11:**
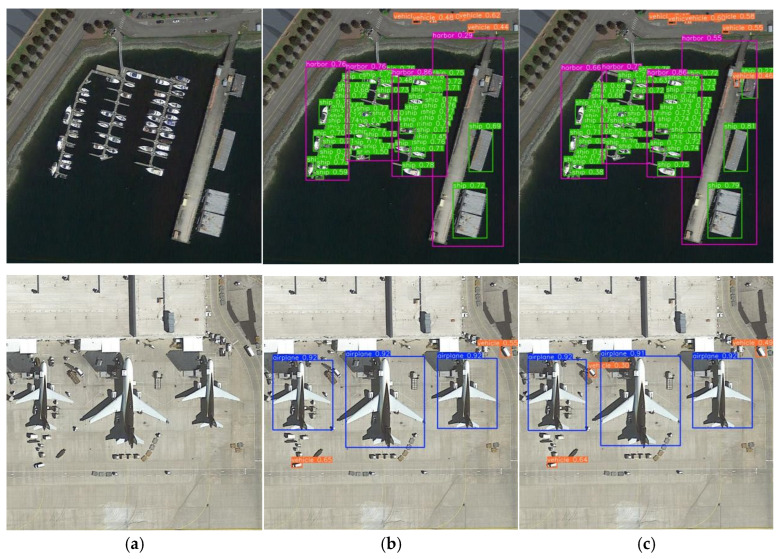
Visualization results of different models for the DIOR dataset: (**a**) original image, (**b**) YOLOv8n, (**c**) HSF-YOLO.

On the DIOR dataset, HSF-YOLO achieves higher precision and recall compared with baseline models, achieving a precision of 89.6%, recall of 82.6%, mAP@0.5 of 88.7%, and mAP@0.5:0.95 of 66.6%. Compared with the baseline YOLOv8n, HSF-YOLO achieves respective gains of 1.2%, 2.4%, 2.3%, and 2.5%. When compared with traditional detectors and representative YOLO models, HSF-YOLO exhibits clear advantages in both precision and recall, highlighting the comprehensive performance gains brought by its architectural improvements. When evaluated against a range of recent advanced detection models including MTGS-Yolo, CoF-Net, YOLO-GE, GLFE-YOLOX, YOLO-SPP, and RNAF-YOLO, HSF-YOLO still leads in overall performance. In particular, it surpasses the strongest competitor, GLFE-YOLOX, by 1.4% in mAP@0.5:0.95, and also outperforms it by 0.8% and 1.1% in precision and recall, respectively. In addition, DETR-based models such as DINO and PSWP-DETR achieve mAP@0.5:0.95 scores of 60.5% and 63.1%, respectively. Although they rely on the strong representation capabilities of the Transformer architecture, their overall performance still falls short of that of HSF-YOLO. These results emphasize the significant effectiveness of the proposed multi-module fusion strategy in feature extraction and semantic aggregation, especially in terms of robustness to small-scale object detection under complex conditions. The visualization of results on this dataset is shown in Figure 11.

**Figure 12:**
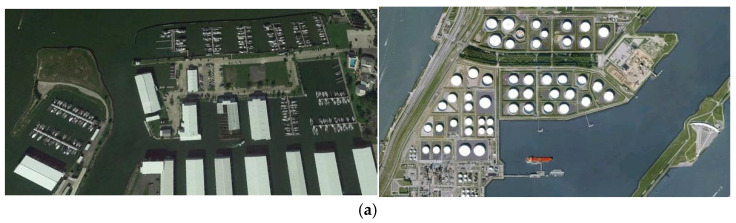

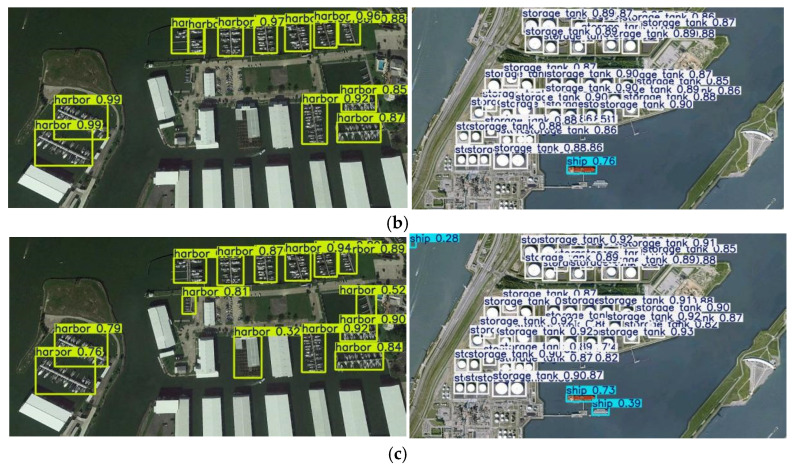
Visualization results of different models for the NWPU VHR-10 dataset: (**a**) original image, (**b**) YOLOv8n, (**c**) HSF-YOLO.

Similarly, on the NWPU VHR-10 dataset, HSF-YOLO continues to maintain a leading position, achieving precision, recall, mAP@0.5, and mAP@0.5:0.95 values of 93.3%, 89.3%, 93.0%, and 62.1%, respectively. Compared with YOLOv8n, the model achieves improvements of over 2% across all metrics, reflecting strong generalization and stability. Traditional detectors such as SSD and Faster R-CNN perform poorly on this dataset, further revealing their limitations for SOD in complex remote sensing environments. Compared with more recent advanced models like GLFE-YOLOX, SOD-YOLOv10, YOLO-GE, FPSOD, SFSANet, and YGNet, HSF-YOLO not only achieves mAP@0.5:0.95 improvements ranging from 0.2% to 1.9% but also maintains slight advantages in precision and recall. In addition, two DETR-based detectors, DFS-DETR and EMSD-DETR, achieve mAP@0.5:0.95 scores of 60.3% and 60.1%, respectively. Although they benefit from the modeling strength of Transformer architectures and achieve relatively high mAP@0.5 scores (90.7% and 90.2%), their overall performance still falls behind that of HSF-YOLO. These results indicate HSF-YOLO enhances detection accuracy while simultaneously improving object coverage and recall performance, underscoring the strong adaptability and robustness of its multi-scale feature fusion and attention mechanism design in diverse remote sensing object detection scenarios. The visualization results on this dataset are shown in Figure 12.

Collectively, these comparative results across three diverse and challenging datasets validate the robust generalization ability and superior detection capability of HSF-YOLO. The substantial gains over both baseline and state-of-the-art methods confirm the effectiveness of our proposed architectural enhancements, particularly in scenarios characterized by small object sizes, scale variance, occlusion, and complex backgrounds.

### 4.5. Ablation Experiments

To comprehensively evaluate the effectiveness of each proposed module in the HSF-YOLO framework, ablation experiments are conducted on the VisDrone2019, DIOR, and NWPU VHR-10 datasets. Specifically, we assess the impact of four modules: the high-resolution P2 detection layer, the HAEC module for multi-scale context extraction, the C2f_SIS attention module for enhanced spatial-channel feature fusion, and the FGR-Loss, a novel loss function designed to refine regression precision. These modules are incrementally integrated into the YOLOv8n baseline, and results are presented in Table 4, Table 5 and Table 6.

The ablation experiments conducted on the VisDrone2019 dataset demonstrate that the baseline YOLOv8n model achieves a precision (P), recall (R), mAP@0.5, and mAP@0.5:0.95 of 46.1%, 35.1%, 35.3%, and 20.6%, respectively (Table 4). Introducing the P2 detection layer alone increases mAP@0.5:0.95 to 22.0% (+1.4%) and recall to 37.5% (+2.4%). The HAEC module, when used independently, further enhances semantic understanding, resulting in mAP@0.5 and mAP@0.5:0.95 of 36.5% and 21.6%, respectively; when combined with P2, the mAP@0.5:0.95 is improved to 23.6%. Replacing the standard C2f modules with the proposed C2f_SIS improves precision to 48.2% and raises mAP@0.5:0.95 to 22.1%, indicating enhanced capability in spatial and channel-wise feature fusion. The FGR-Loss module, when introduced alone, improves mAP@0.5:0.95 from 20.6% to 21.0%. When P2, HAEC, and C2f_SIS are combined, the model performance is significantly improved, achieving 49.5% precision, 38.7% recall, 38.3% mAP@0.5, and 23.6% mAP@0.5:0.95, demonstrating strong complementarity among these components. Further introducing FGR-Loss on top of this configuration boosts mAP@0.5:0.95 to 24.1%. Ultimately, with all modules integrated, the model achieves the best overall performance, reaching 51.6% precision, 40.0% recall, 41.0% mAP@0.5, and 24.6% mAP@0.5:0.95, with 3.4 M parameters and 17.1 GFLOPs. These results indicate that the proposed modules are not only effective when applied individually but also exhibit synergy when combined.

On the DIOR dataset (Table 5), the baseline achieves P, R, mAP@0.5, and mAP@0.5:0.95 of 88.4%, 80.2%, 86.4%, and 64.1%, respectively. The introduction of the P2 detection layer alone increases the mAP@0.5 from 86.4% to 86.9% (+0.5%) and the mAP@0.5:0.95 from 64.1% to 64.5% (+0.4%). When the HAEC module is added independently, the model achieves a recall of 80.9%. When HAEC is combined with the P2 layer, the mAP@0.5:0.95 further improves to 66.1%, with precision and recall reaching 89.5% and 81.6%, respectively. Replacing the original C2f module with the proposed C2f_SIS module increases the precision to 89.0%, mAP@0.5 to 87.2%, and mAP@0.5:0.95 to 65.3%. When this module is used in combination with P2 and HAEC, all performance metrics show steady improvements. The FGR-Loss module, when introduced independently, boosts the mAP@0.5:0.95 from 64.1% to 64.9% with almost no additional computational cost; when added to the P2, HAEC and C2f_SIS configuration, it increases the recall to 82.0% and the mAP@0.5 to 88.2%. Ultimately, integrating all modules yields the best performance, achieving a precision of 89.6%, recall of 82.6%, mAP@0.5 of 88.7%, and mAP@0.5:0.95 of 66.6%. These results fully demonstrate the individual effectiveness of each module as well as the significant synergistic gains achieved when they are used together.

On the NWPU VHR-10 dataset (Table 6), the baseline model records precision, recall, mAP@0.5, and mAP@0.5:0.95 of 91.1%, 86.6%, 90.7%, and 60.0%. After independently introducing the P2 detection layer, the precision increased to 92.3%, recall reached 87.2%, mAP@0.5 rose to 91.2%, and mAP@0.5:0.95 improved to 60.6%. When the HAEC module was added alone, all metrics increased by 0.4%. With the inclusion of the C2f_SIS module, precision rose to 92.0%, mAP@0.5 increased to 91.5%, recall reached 87.3%, and mAP@0.5:0.95 improved to 61.0%. The standalone introduction of FGR-Loss led to an increase in mAP@0.5 to 91.2%. When P2, HAEC, and C2f_SIS were used together, recall significantly improved to 88.6%, and mAP@0.5 reached 92.4%, while precision remained stable at 92.0%, demonstrating strong synergistic gains. With the addition of C2f_SIS, recall further increased to 89.4%, and mAP@0.5 and mAP@0.5:0.95 reached 92.8% and 61.7%, respectively. When all modules were ultimately integrated, the model achieved its highest precision of 93.3%, recall of 89.3%, and mAP@0.5 and mAP@0.5:0.95 improved to 93.0% and 62.1%, respectively. Each module demonstrated significant contributions in enhancing precision, recall, and localization accuracy, and their combined use resulted in stable and remarkable performance improvements.

**Figure 13:**
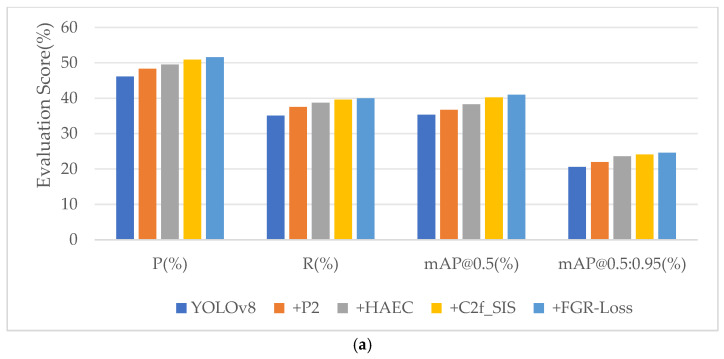

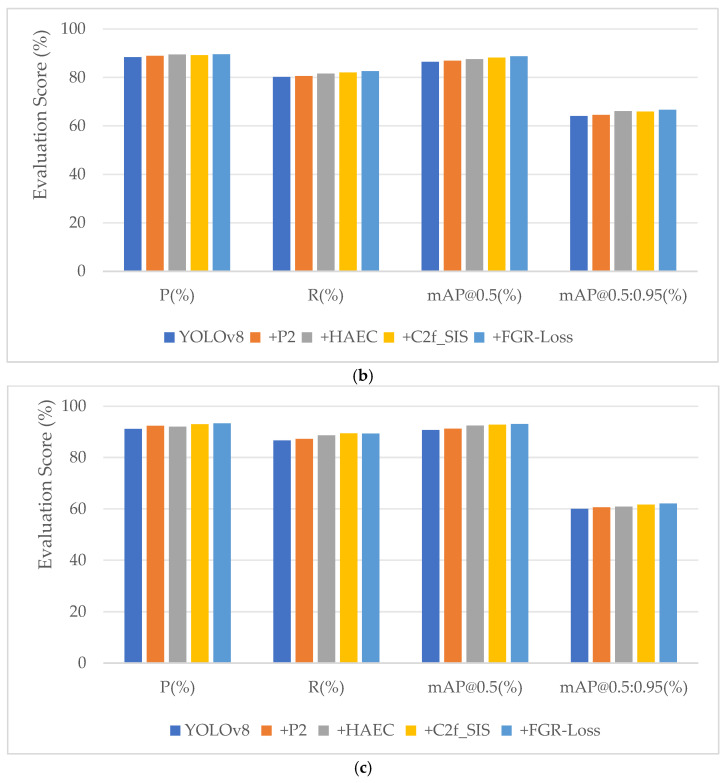
The impact of different module combinations on the detection performance of the HSF-YOLO model: (**a**) VisDrone2019 dataset, (**b**) DIOR dataset, (**c**) NWPU VHR-10 dataset.

In summary, the ablation results across the three datasets indicate that each proposed module contributes positively to the model’s performance. The high-resolution P2 detection layer effectively enhances small-object detection, the HAEC module improves multi-scale contextual understanding, the C2f_SIS module strengthens spatial-channel feature fusion, and the FGR-Loss provides a relatively significant improvement in localization precision and training stability. The impact of different module combinations on the detection performance of the HSF-YOLO model on three datasets is shown in Figure 13. Overall, the combined effect of these modules facilitates performance gains of the HSF-YOLO framework across multiple datasets.

## 5. Conclusions

In this paper, a robust and unified detection framework HSF-YOLO is proposed for SOD in RSIs. The proposed method addresses key challenges such as weak feature representation, scale variation, object occlusion, and background interference, which commonly lead to missed and false detections in dense and complex remote sensing scenes. To enhance multiscale perception and semantic consistency, we introduced the HAEC module, which simultaneously captures global context and preserves fine-grained spatial information. To improve attention interaction and feature discrimination, we proposed the C2f_SIS module, incorporating spatial and enhanced channel attention with a shuffle mechanism. Furthermore, we designed the FGR-Loss to dynamically emphasize low-IoU and ambiguous samples, promoting better localization precision and reducing prediction overlap in dense object regions. Experimental results on three benchmark remote sensing datasets—DIOR, VisDrone2019, and NWPU VHR-10—demonstrate that the proposed HSF-YOLO framework outperforms the baseline YOLOv8 model. Specifically, it achieves improvements of 5.7% and 4.0% in mAP@0.5 and mAP@0.5:0.95 on the VisDrone2019 dataset, 2.3% and 2.5% on the DIOR dataset, and 2.3% and 2.1% on the NWPU VHR-10 dataset, respectively. These quantitative results clearly validate the superiority of HSF-YOLO in terms of detection accuracy and robustness for small, occluded, and scale-varying objects in complex remote sensing scenarios. Despite the notable performance gains, the current model still faces challenges in inference efficiency and deployment cost, particularly on resource-constrained edge computing devices. Future work will focus on optimizing and compressing the model architecture to enhance its deployment capability and real-time detection performance on embedded platforms.

## Figures and Tables

**Table 1:** The results of different base models on the VisDrone2019 dataset.

Model	P (%)	R (%)	mAP@0.5 (%)	mAP@0.5:0.95 (%)
SSD [47]	-	-	25.6	13.6
Faster R-CNN [15]	-	-	19.7	10.7
YOLOv3 [20]	43.6	33.5	32.9	18.9
YOLOv5s [21]	45.4	34.7	34.2	19.7
YOLOX [22]	45.8	35.0	34.9	19.6
YOLOv8n	46.1	35.1	35.3	20.6
DFS-DETR [48]	50.4	39.2	39.3	23.1
EMST-DETR [45]	49.1	38.6	38.8	23.0
Wang et al. [23]	49.3	38.5	38.9	22.1
Nei et al. [30]	48.5	38.6	38.1	22.3
SCM-YOLO [25]	47.5	37.2	36.9	21.8
MTGS-Yolo [42]	48.2	38.9	38.3	22.5
YOLO-GE [10]	49.0	39.8	40.2	23.1
FPSOD [49]	49.3	38.2	38.6	23.1
TA-YOLO [50]	50.1	39.3	39.9	24.0
RNAF-YOLO [37]	50.4	39.8	39.4	23.7
MFF-YOLOv8 [29]	50.6	39.6	40.1	23.5
Ours	51.6	40.0	41.0	24.6

**Table 2:** The results of comparative experiments on the DIOR dataset.

Model	P (%)	R (%)	mAP@0.5 (%)	mAP@0.5:0.95 (%)
SSD [47]	-	-	69.2	48.3
Faster R-CNN [15]	-	-	67.9	46.5
YOLOv3 [20]	79.6	70.5	73.4	58.7
YOLOv5s [21]	82.7	74.6	78.3	60.0
YOLOX [22]	83.6	78.2	82.0	61.7
YOLOv8n	88.4	80.2	86.4	64.1
DINO [46]	86.6	79.5	82.2	60.5
PSWP-DETR [51]	87.5	80.2	84.9	63.1
MTGS-Yolo [42]	84.6	78.2	82.6	56.8
CoF-Net [40]	88.3	80.2	85.9	64.5
YOLO-GE [10]	87.9	80.8	85.6	63.6
GLFE-YOLOX [27]	88.8	81.5	88.1	65.2
YOLO-SPP [33]	87.7	81.5	87.3	64.7
RNAF-YOLO [37]	88.1	81.6	87.5	64.7
Ours	89.6	82.6	88.7	66.6

**Table 3:** Experimental results of comparative experiments on the NWPU VHR-10 dataset.

Model	P (%)	R (%)	mAP@0.5 (%)	mAP@0.5:0.95 (%)
SSD [47]	-	-	75.3	42.4
Faster R-CNN [15]	-	-	79.8	45.1
YOLOv3 [20]	86.2	81.5	86.7	53.3
YOLOv5s [21]	89.5	84.2	90.2	58.0
YOLOX [22]	90.1	85.1	90.3	59.8
YOLOv8n	91.1	86.6	90.7	60.0
DFS-DETR [48]	92.5	88.2	90.7	60.3
EMST-DETR [45]	91.6	87.5	90.2	60.1
GLFE-YOLOX [27]	90.8	86.0	90.9	59.8
SOD-YOLOv10 [43]	92.7	88.2	92.5	61.3
YOLO-GE [10]	90.8	85.7	91.1	59.3
YGNet [31]	91.8	87.8	91.8	60.2
FPSOD [49]	92.9	88.7	92.8	61.9
SFSANet [39]	93.1	89.0	92.3	61.8
Ours	93.3	89.3	93.0	62.1

**Table 4:** The ablation experiment results of our algorithm modules on the VisDrone2019 dataset.

YOLOv8	P2	HAEC	C2f_SIS	FGR-Loss	P (%)	R (%)	mAP@0.5 (%)	mAP@0.5:0.95(%)	Params(M)	FLOPs (G)
√					46.1	35.1	35.3	20.6	3.0	8.1
√	√				48.3	37.5	36.7	22.0	3.1	12.2
√		√			47.2	36.0	36.5	21.6	3.0	12.0
√			√		48.2	37.1	37.7	22.1	3.3	9.1
√				√	46.6	35.5	35.8	21.0	3.0	8.1
√	√	√			49.5	38.7	38.3	23.6	3.1	16.1
√	√	√	√		50.9	39.6	40.2	24.1	3.4	17.1
√	√	√	√	√	51.6	40.0	41.0	24.6	3.4	17.1

**Table 5:** The ablation experiment results of our algorithm modules on the DIOR dataset.

YOLOv8	P2	HAEC	C2f_SIS	FGR-Loss	P (%)	R (%)	mAP@0.5 (%)	mAP@0.5:0.95 (%)
√					88.4	80.2	86.4	64.1
√	√				88.8	80.6	86.9	64.5
√		√			88.6	80.9	86.7	64.2
√			√		89.0	81.1	87.2	65.3
√				√	88.6	80.5	87.0	64.9
√	√	√			89.5	81.6	87.5	66.1
√	√	√	√		89.2	82.0	88.2	65.9
√	√	√	√	√	89.6	82.6	88.7	66.6

**Table 6:** The ablation experiment results of our algorithm modules on the NWPU VHR-10 dataset.

YOLOv8	P2	HAEC	C2f_SIS	FGR-Loss	P(%)	R(%)	mAP@0.5(%)	mAP@0.5:0.95(%)
√					91.1	86.6	90.7	60.0
√	√				92.3	87.2	91.2	60.6
√		√			91.5	87.0	91.1	60.4
√			√		92.0	87.3	91.5	61.0
√				√	91.3	87.0	91.2	60.3
√	√	√			92.0	88.6	92.4	60.9
√	√	√	√		92.9	89.4	92.8	61.7
√	√	√	√	√	93.3	89.3	93.0	62.1

## Data Availability

Data will be provided by the corresponding author upon reasonable request by the reader.
